# Voices from the Frontline: Healthcare Workers’ Perspectives on Government Health Reforms in Iran

**DOI:** 10.34172/aim.34341

**Published:** 2025-07-01

**Authors:** Ali Dabbagh, Firoozeh Madadi, Mina Fakhrzadegan

**Affiliations:** ^1^Department of Anesthesiology, Shahid Modarres Hospital, School of Medicine, Shahid Modarres Hospital, Shahid Beheshti University of Medical Sciences, Tehran, Iran; ^2^Anesthesiology Research Center, Shahid Beheshti University of Medical Sciences, Tehran, Iran; ^3^Department of Anesthesiology, Ayatollah Taleghani Hospital, School of Medicine, Shahid Beheshti University of Medical Sciences, Tehran, Iran; ^4^Department of Orthopedics, Taleghani Hospital Clinical Research Development Unit, Shahid Beheshti University of Medical Sciences, Tehran, Iran

**Keywords:** Developing countries, Healthcare reform, Health personnel, Health policy, Medical education, Workforce

## Abstract

**Background:**

The healthcare system is a crucial indicator of government performance, especially in developing countries like the Islamic Republic of Iran, which has seen significant reforms in recent decades. This study explores healthcare workers’ perceptions of recent government health policies.

**Methods:**

This study was conducted as a national survey. An anonymous questionnaire containing 22 key challenges facing the healthcare sector was distributed via social media to healthcare employees. The aim was to gather insights on the challenges confronting Iran’s healthcare system. After collecting the responses, we evaluated and analyzed the data.

**Results:**

Over one week, we received responses from 805 healthcare workers. The findings revealed that the following issues were considered as the most important priorities across various categories: the healthcare system’s entitlement from the gross national product; addressing the problems of doctors, including their income; preventing migration; combating non-scientific health practices; the challenges of medical education for future healthcare providers (such as reduced career hopes, desires to emigrate, drug use, and mental health issues like suicide); and tackling corruption in the country’s pharmaceutical market.

**Conclusion:**

The most significant challenges identified were economic issues and the medical education process, from admission to graduation. Notably, as people’s ages increased, the scores related to economic challenges and job burnout also rose.

## Introduction

 The healthcare system is a critical measure of a government’s performance. Over the past few decades, the Islamic Republic of Iran, as a developing country, has undergone significant reforms.^1,2^ One of the most notable advancements has been improving the primary healthcare system, which has focused on enhancing accessibility, comprehensiveness, coordination, community involvement, health promotion, disease prevention, and early detection and management. Additionally, there have been improvements in medical education, research, and the introduction of new treatment modalities.^3,4^

 However, some critical issues persist, such as low wages for nurses and junior physicians and burnout among healthcare workers. Furthermore, the healthcare system appears to be increasingly vulnerable due to the severe international sanctions imposed on Iran.^2,5^

 This study examines how healthcare workers perceive the government’s recent health policies, particularly those they consider most impactful. It will illuminate healthcare professionals’ priorities, concerns, and expectations regarding these reforms and discuss their implications for the healthcare system.^6,7^ Despite the progress made, the healthcare system faced new challenges during the COVID-19 pandemic and existing issues.^8-11^ On the other hand, the government underwent an unexpected change following the President’s helicopter crash on May 19, 2024, and is working diligently to expedite its efforts.

 While health system policies often aim to address systemic challenges, they frequently lack integration of insights from frontline healthcare workers, who are the ones who carry out these policies firsthand and witness their effects. This study uniquely focuses on healthcare workers’ viewpoints, providing a grassroots-level understanding of the challenges and priorities within Iran’s national healthcare system.

## Material and Methods

 We conducted an anonymous online questionnaire survey on social media targeting healthcare workers to gather insights about the challenges facing Iran’s healthcare system. Our study was designed to capture voices that are underrepresented in current health system evaluations but can significantly affect it.

 The questionnaire addressed 22 of the most pressing issues and received approval from the Ethics Committee and the Vice Chancellor of Research at Shahid Beheshti University of Medical Sciences (SBMU) in Tehran, Iran, under the ethics code IR.SBMU.REC.1403.13. The questions included in the survey are listed in Table 1. Additional questions about the participant’s age, gender and job status were also included (Table 2). IRB waived the requirement for informed written consent due to the study method.

**Table 1:** Healthcare System Challenges or Priorities Addressed in the Questionnaire

**Question No.**	**Challenges with priority from the most important to the least**
1	Common medical education problems for learners including students and assistants (such as quality of education, job fatigue, monthly salaries of assistants and interns...)
2	Problems of future medical education for learners, including students and assistants (such as reduced hope for a future career, desire to emigrate, smoking, drugs, suicide...)
3	Changing the eligibility criteria of students in universities of medical science
4	Improving the infrastructure of the network system in cities with a population of over 200,000 people
5	Improving the infrastructure of the network system in cities with a population of less than 200,000 people
6	Justice-oriented and concentrated on offering poor and deprived provinces health services
7	Focus on the prevention of emerging infectious diseases and chronic diseases
8	Education of the society at the level of the general public in the field of health
9	Preparation and implementation of artificial intelligence infrastructure in the health system
10	Emphasis on strengthening the scientific process in the expansion of health system research and technology
11	Focus on identifying the social components affecting the mental health of Iran's 88 million population
12	Expansion and strengthening of health tourism due to its rapid expansion in neighboring countries
13	Preparation for critical situations such as natural disasters due to the country's proneness to accidents
14	Focusing on the reconstruction of the country's pharmaceutical industry and injecting liquidity into pharmaceutical factories
15	Fighting against potential and actual corruption in the pharmaceutical market of the country
16	Entitlement of the health system from the gross national product
17	Full integration of basic insurance organizations in the Ministry of Health
18	Full supervision of the Ministry of Health on supplementary health insurances
19	Fighting against non-scientific methods in health
20	Combating medical violations in the health system
21	Focusing on solving the problems of doctors, including their income and preventing migration
22	Focusing on solving the problems of the nursing system, including the income and prevention of immigration

The participants were asked about the priority of each issue by rating them from 1 to 10. (Priority 1 implied the lowest priority, and priority 10 denoted the most important issue.)

**Table 2:** Characteristics of the Study Population

**Characteristics**	**Case (N=807)**	
Age (year), mean ± SD (Range)	48.1 ± 10.7 (Range: 19‒79)	
Gender, No. (%)	Male	523 (64.8)
	Female	284 (35.2)
Healthcare management experience, No. (%)	Yes	419 (51.9)
	No	388 (48.1)
Job Status, No. (%)	Government	323 (40)
	Private Sector	173 (21.4)
	Both	311 (38.6)
Educational major, No. (%)	Clinical Medicine	488 (60.5)
	Other Majors	317 (39.5)
City of employment, No. (%)	Tehran	425 (52.7)
	Other Cities	382 (47.3)
Work experience abroad, No. (%)	Yes	94 (11.5)
	No	713 (88.5)

 The questionnaire’s link was shared on various social media groups, including the official telegram channel of the medical Council of the Islamic Republic of Iran. The survey was done in a period of one week, and the collected data was analyzed afterward.

###  Statistical Analysis

 Statistical analysis was conducted using the R software version 4.3.0. Quantitative data were described by mean, standard deviation, and range, while percentages were used to describe categorical data.

## Results

 Out of 807 participants, there were 523 males (64.8%), and 284 (35.2%) females, with a mean age of 48.1 years ( ± 10.7, Range: 19‒79). Among the participants, 419 (51.9%) had experience in healthcare management, with an average of 10.64 years ( ± 8.54, Range: 1‒40). Most participants, 488 individuals (60.5%), had a background in clinical medicine, and more than half were employed in Tehran, the capital. Only 94 participants (11.5%) reported having work experience abroad (see Table 2).

**Figure 1:**
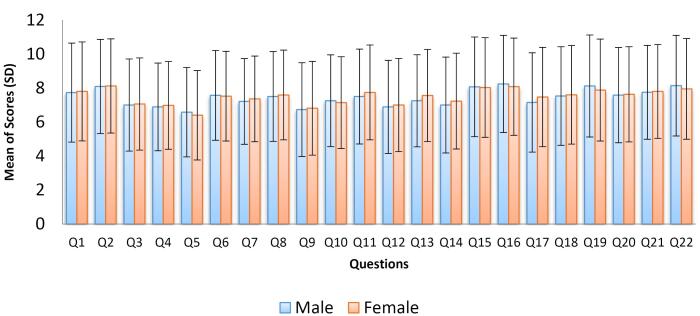
Comparison of Answers to Questions 1‒22 Between Different Gender Groups

 In just over a week, we received responses from 805 healthcare workers out of more than 340 000. As shown in Figure 1, the results indicate no statistically significant difference between the average scores of the 22 priorities based on gender. However, five specific items emerged as the most important priorities for both women and men. These items are:

1. The entitlement of the health system from the gross national product
2. Focusing on solving problems faced by doctors, including their income and the prevention of migration
3. Addressing non-scientific methods in healthcare
4. Tackling issues related to the future of medical education for learners, including students and assistants, such as decreased career prospects, the desire to emigrate, and challenges like smoking, drug use, and suicide
5. Combatting potential and actual corruption in the country’s pharmaceutical market.

**Figure 2:**
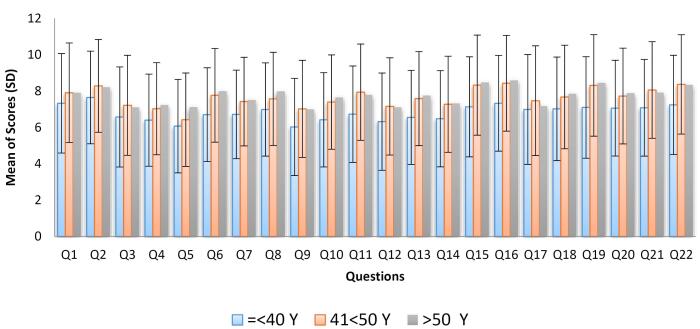
Comparison of Answers to Questions 1‒22 Between Different Age Groups

 An analysis of the average scores for 22 hypothetical priority items in the health system, categorized by age group, revealed that the prioritization of 21 questionnaire items showed a significant relationship with the respondents’ ages. However, priority item 17, “Full integration of basic insurance organizations in the Ministry of Health,” did not show significant differences between the age groups (see Figure 2).

 Additionally, analyzing the average scores of 22 hypothetical priority items within the health system across different provinces revealed several key areas of concern. The highest priorities identified by all respondents, regardless of whether they lived in Tehran or another city, included:

1. Combating both potential and actual corruption in the pharmaceutical market.
2. Allocating a portion of the gross national product to support the health system.
3. Addressing challenges in future medical education for learners, such as diminishing career prospects, a desire to emigrate, and issues related to smoking, drug use, and suicide.
4. Focusing on resolving problems faced by doctors, particularly concerning their income.
5. Preventing the migration of healthcare professionals.
6. Opposing non-scientific methods in health practices.

These findings were also consistently observed when evaluating the average scores of the 22 priority items by different academic fields.

## Discussion

 This study provides a unique opportunity for policymakers to align systemic reforms with the real-world experiences of healthcare workers. By addressing the issues raised by healthcare workers (who are the backbone of healthcare delivery), policies can achieve greater relevance, effectiveness, and sustainability.

 The survey findings highlight that the most pressing issues are economic challenges and the medical education process, from admission to graduation. Urgent government action is needed to address the effects of low wages and burnout among healthcare workers. Older respondents reported higher scores on questions related to economic factors and burnout.

 Our results align closely with the findings of Doshmangir et al, who indicated workforce dissatisfaction and lack of adequate wages as persistent challenges, as well as inefficient resource allocation.^12^ The workforce dissatisfaction and economic issues they addressed is consistent with our findings. However, our study adds depth by directly approaching the healthcare workers’ perception of these deficiencies.

 Combating both potential and actual corruption in the pharmaceutical market is also a critical challenge. Corruption can manifest in various forms, including manipulation in procurement processes, lack of transparency in pricing, and undue influence in regulatory decisions. Specifically in Iran, a qualitative policy analysis by Ebadi Fardazar et al identified structural challenges that may contribute to corruption risks, such as inefficient managerial practices, inadequate policy implementation, and lack of alignment with international standards in areas like procurement, supply chain management, and research and development.^13^

 These systemic issues not only hinder the performance of Iran’s pharmaceutical sector but also create conditions where corruption can flourish. Addressing these vulnerabilities requires strategic reforms, including enhanced transparency,^14^ digitized tracking systems, and the alignment of national regulations with international best practices.^15^

 Khankeh et al also emphasized structural and policy advancements of our healthcare system over the past three decades. Like our study, they identified a gap between high-level policy goals and ground-level realities.^1^ However, they focused on structural goals rather than frontline challenges. In this study, we emphasized some critical barriers which may explain the possible failure of policy goals. The most important barriers according to our study are migration, economic constraints, and dissatisfaction of healthcare workers.

 Yazdi-Feyzabadi and colleagues used the 3i framework to assess Iran’s District Health Network Policy, examining the interplay of ideas, interests, and institutions in policymaking. They concluded that a better alignment of ideas (policy goals) with interests (stakeholder priorities) could improve outcomes, which also highlights institutional inefficiencies and lack of stakeholder participation as obstacles to policy effectiveness.^16^ They emphasized the importance of stakeholder involvement; however, unlike our study, they addressed institutions rather than workers.

 Despite the global rise of artificial intelligence, it is surprising to see its low implementation scores. The government must prioritize and tackle this issue, as well. The limited implication of AI in developing countries is mostly due to limited infrastructure, lack of high-quality data, shortage of professionals and financial constraints.^17,18^ Addressing these challenges requires a quick multifaceted approach. Additionally, it is essential to establish clear priorities to effectively evaluate future advancements in our national healthcare system.

 From a political perspective, our findings highlight the effectiveness of initiatives to improve public health. In democratic countries, governments tend to be more accountable and responsible for their responsibilities. Consequently, in democratic nations, the inefficiency in implementing health programs is generally lower, which leads to more successful public health policies. It is important to note that previous assessments indicate that this success is particularly pronounced in democratic countries with a desirable level of economic development and per-capita income.^19-22^

 According to previous studies, in poorer countries with democratic policies, the government’s efforts to maintain its position have often led to economic corruption, subsequently decreasing the efficiency of the public health system.^19,21,22^ This issue is more prevalent in democratic countries with low per-capita incomes, where the government has limited direct access to tax revenues. Supporting this analysis, some studies have demonstrated that the economic systems of wealthier democratic countries tend to be more honest and successful.^23-25^

 In addition to their crucial role, donors can significantly improve the structures and tools available within the public health system. Effective communication between the health system and democratic institutions is essential for health policymaking and developing the most effective guidelines. Based on our theoretical model and previous studies, these findings can be discussed in broader policy circles to enhance the efficiency of the health system.^26,27^

 According to our study, despite many advancements achieved in our healthcare system, there are still significant concerns, most of which are attributable to economic challenges which should be considered to avoid further dissatisfaction of healthcare workers. Policymakers may take measures to fulfill the interests of its human resources.

## Limitations

 The sample size is relatively small, compared to the whole population of Iran’s healthcare workers, which is estimated to be about 340,000. Therefore, we propose that policymakers conduct a survey with a higher sample size. Also, a brief survey of healthcare workers might be a good initiative to give feedback to policy makers but is not enough and more surveys might be done to achieve the high priority challenges. Moreover, after identifying the challenges, frontline voices may have wise solutions for some of these issues and their role must not be underestimated.

## Conclusion

 The primary concerns identified were the economic challenges and the medical education process from admission to graduation. It is crucial to note that the scores related to economic issues and job burnout tend to rise as people age. There is an urgent need for government action to address the effects of low wages and burnout among healthcare workers.
